# Free-Living, Psychrotrophic Bacteria of the Genus *Psychrobacter* Are Descendants of Pathobionts

**DOI:** 10.1128/mSystems.00258-21

**Published:** 2021-04-13

**Authors:** Daphne K. Welter, Albane Ruaud, Zachariah M. Henseler, Hannah N. De Jong, Peter van Coeverden de Groot, Johan Michaux, Linda Gormezano, Jillian L. Waters, Nicholas D. Youngblut, Ruth E. Ley

**Affiliations:** a Department of Microbiome Science, Max Planck Institute for Developmental Biology, Tübingen, Germany; b Department of Biology, Queen’s University, Kingston, Ontario, Canada; c Conservation Genetics Laboratory, University of Liège, Liège, Belgium; d Centre de Coopération Internationale en Recherche Agronomique pour le Développement (CIRAD), UMR ASTRE, Montpellier, France; e Department of Vertebrate Zoology, American Museum of Natural History, New York, New York, USA; University of California San Diego

**Keywords:** genomics, phylogeny, *Psychrobacter*, psychrophiles

## Abstract

Host-adapted microorganisms are generally assumed to have evolved from free-living, environmental microorganisms, as examples of the reverse process are rare. In the phylum *Gammaproteobacteria*, family *Moraxellaceae*, the genus *Psychrobacter* includes strains from a broad ecological distribution including animal bodies as well as sea ice and other nonhost environments. To elucidate the relationship between these ecological niches and *Psychrobacter*’s evolutionary history, we performed tandem genomic analyses with phenotyping of 85 *Psychrobacter* accessions. Phylogenomic analysis of the family *Moraxellaceae* reveals that basal members of the *Psychrobacter* clade are *Moraxella* spp., a group of often-pathogenic organisms. *Psychrobacter* exhibited two broad growth patterns in our phenotypic screen: one group that we called the “flexible ecotype” (FE) had the ability to grow between 4 and 37°C, and the other, which we called the “restricted ecotype” (RE), could grow between 4 and 25°C. The FE group includes phylogenetically basal strains, and FE strains exhibit increased transposon copy numbers, smaller genomes, and a higher likelihood to be bile salt resistant. The RE group contains only phylogenetically derived strains and has increased proportions of lipid metabolism and biofilm formation genes, functions that are adaptive to cold stress. In a 16S rRNA gene survey of polar bear fecal samples, we detect both FE and RE strains, but in *in vivo* colonizations of gnotobiotic mice, only FE strains persist. Our results indicate the ability to grow at 37°C, seemingly necessary for mammalian gut colonization, is an ancestral trait for *Psychrobacter*, which likely evolved from a pathobiont.

**IMPORTANCE** Host-associated microbes are generally assumed to have evolved from free-living ones. The evolutionary transition of microbes in the opposite direction, from host associated toward free living, has been predicted based on phylogenetic data but not studied in depth. Here, we provide evidence that the genus *Psychrobacter*, particularly well known for inhabiting low-temperature, high-salt environments such as sea ice, permafrost soils, and frozen foodstuffs, has evolved from a mammalian-associated ancestor. We show that some *Psychrobacter* strains retain seemingly ancestral genomic and phenotypic traits that correspond with host association while others have diverged to psychrotrophic or psychrophilic lifestyles.

## INTRODUCTION

Association with vertebrate hosts was shown to be the largest factor driving differences in the 16S rRNA diversity of microbiomes sampled globally (1, 2). Recent analysis of metagenome-assembled genomes from multiple habitats shows that many are either enriched in animal hosts or the environment but generally not both (3). Specialization to host association can also be seen at higher taxonomic levels, indicating that whole lineages may have diverged once animal hosts were first successfully colonized. For instance, within the *Bacteroidetes*, the taxa that are mammalian gut associated are derived from phylogenetically basal clades that include free-living and invertebrate-associated taxa (4). These patterns of distribution imply that specialization to the warm animal host habitat is mostly incompatible with fitness in other environments. The specific diet of the host, the chemical environment of the gut, competition with an extremely dense surrounding microbial community, and direct selection by the host immune system all likely contribute to the restricted taxonomy of gut-associated microbes (5).

One line of evidence for the adaptive evolution of gut microbes is the emergence of specialized functions, such as the ability to bind and degrade host-specific nutrient sources like mucin (6), or resistance to bile acids and their salt conjugates (7). Additionally, many gut-associated commensal taxa lack significant environmental reservoirs, due to the divergence in selective pressures between guts and nongut environments (8). In contrast, many gastrointestinal pathogens such as Vibrio cholerae (9) or Yersinia enterocolitica (10) have high fitness within the gut and outside the gut, typically in soil or water. One phylogenetic analysis has indicated that the evolutionary transition from a free-living lifestyle to a host-associated (including pathogenic) lifestyle is more common than the opposite (11).

Species of *Psychrobacter* have been recovered through culture-based and sequence-based methods from a range of animal microbiomes, including the respiratory blow of marine mammals (12); marine mammal skin (13) and guts (14–16); the throats and guts of birds (17, 18) and fish (19); and many nonhost environments such as seawater (20), sea ice (21), marine sediment (22), glacial ice (23), and permafrost soil (24). Some *Psychrobacter* species are capable of causing disease in mammalian hosts (25, 26); however, *Psychrobacter* infections are very rare, and the factors leading to infection are unclear. *Psychrobacter*’s wide range of environmental sources and adaptation to cold temperatures raise the question whether *Psychrobacter* is truly associated with mammalian hosts, or if it is an allochthonous member of mammalian microbiomes. A comparative genomics analysis of 26 *Psychrobacter* spp. and metadata gleaned from public sources revealed differences in cold adaptation of protein coding sequences between warm-host-associated strains and derived marine and terrestrial strains (27). Furthermore, warm-adapted strains were basal in the *rpoB* gene phylogeny, suggesting that *Psychrobacter* evolved from a mesophilic ancestor. These observations make *Psychrobacter* an interesting candidate to assess how phenotype maps onto phylogeny and source of isolation and to probe into the evolutionary history of a genus with a wide habitat range.

Here, we investigated the evolutionary history of the genus *Psychrobacter*, a group of closely related bacteria with a broad environmental distribution. We generated 85 genomes of *Psychrobacter* accessions, which we phenotyped for growth under different temperature and salt conditions. We used a collection of wild polar bear feces collected from across the Canadian Arctic and assessed the presence of *Psychrobacter* ecotypes as a function of bear diet. Finally, we conducted tests with a subset of strains for colonization of the mammal gut using germfree mice. Our phylogenomic results confirm a mesophilic ancestry for *Psychrobacter* and a common ancestor with the genus *Moraxella*. Our phenotyping revealed that overall, *Psychrobacter* tolerates a wide range of salinity, but growth at 37°C divided the accessions into two ecotypes: those that retained the ability to grow at warm temperatures and can colonize mammalian hosts (flexible ecotype [FE]) and those that lost the ability to grow at warmer temperatures and are instead restricted to colder temperatures (restricted ecotype [RE]). A genomic analysis of the two ecotypes showed genome reduction in the FE strains with high transposon copy numbers, and increased biofilm formation capability in RE strains, as well as higher proportions of lipid metabolism genes. We showed that *Psychrobacter* strains that are basal are FE but that FE strains are also phylogenetically interspersed with RE strains, indicating either readaptation to the animal host or retention of the basal traits. Although both FE and RE strains were detected in the feces of wild polar bears, of the subset of *Psychrobacter* strains tested, only FE strains could colonize the germfree mouse gut. Together, our results indicate the genus *Psychrobacter* evolved from a pathobiont ancestor, having largely lost its ability to associate with animals in its adaptation to psychrophilic, nonhost environments.

## RESULTS

### *Psychrobacter* forms a clade whose basal members are of the *Moraxella* genus.

**FIG 1:**
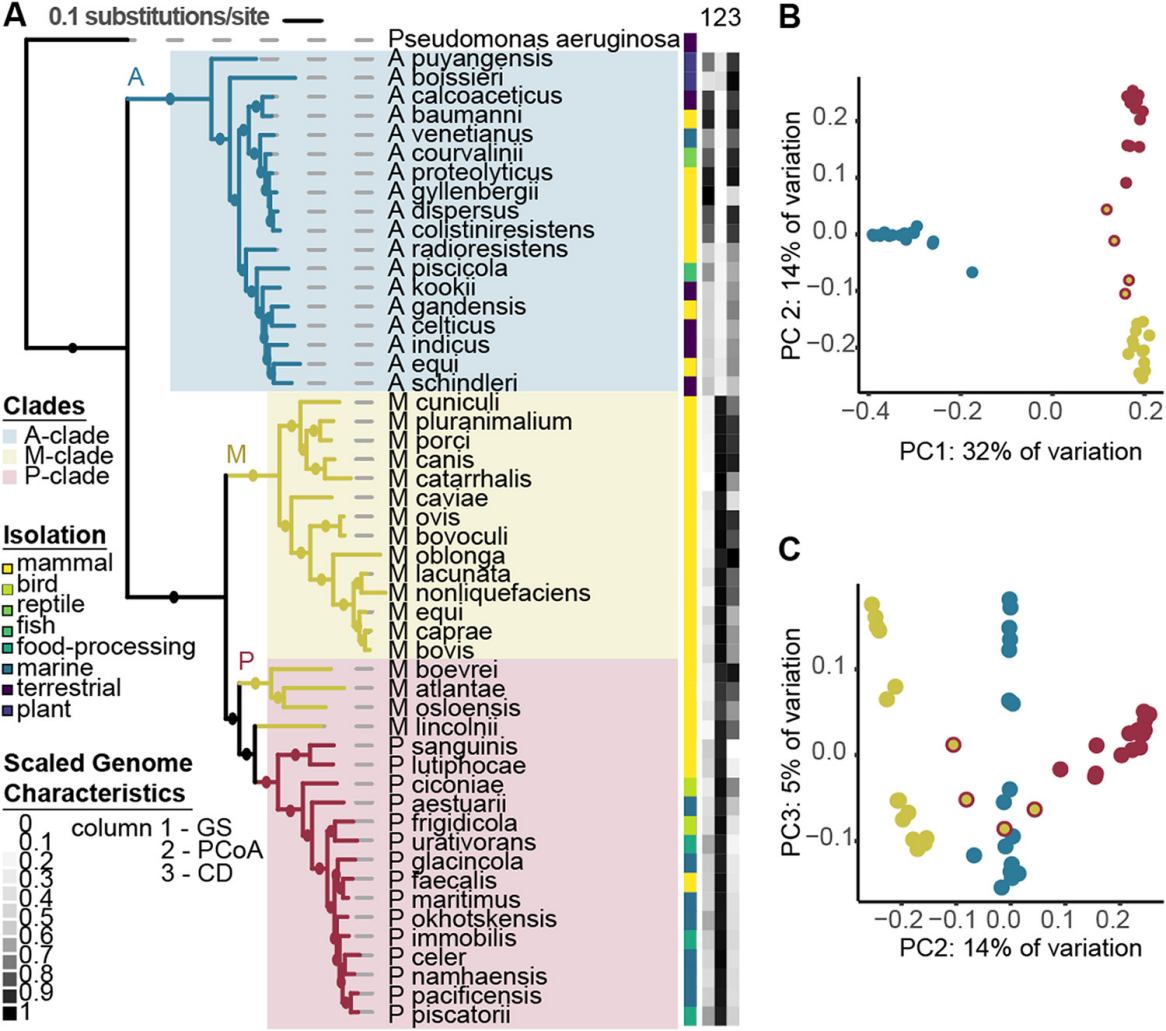
Genomic and phenotypic diversity in the family *Moraxellaceae*. (A) The *Moraxellaceae* family phylogeny was constructed with 51 diverse *Moraxellaceae* genomes using the software PhyloPhlAn, which constructs a phylogeny using FastTree with 1,000 bootstraps, refined by RAxML under the PROTCATG model. Amino acid sequences from 400 marker genes were used in the alignment. Branches with bootstrap support greater than 70% are represented by filled circles. The scale bar represents the average amino acid substitutions per site. Pseudomonas aeruginosa was used as an outgroup. Clades are highlighted in colored blocks, and branches are colored by genus. Isolation source is depicted in a color strip, along with a heatmap of scaled notable genome characteristics that differ between the genera, with 0 representing the smallest value present and 1 the largest value (P. aeruginosa not included). GS, genome size, ranging between 1.8 Mb and 4.5 Mb. PCoA, PC1 values from a PCoA based on gene presence/absence data. CD, genome coding density, ranging from 80% to 89%. Growth temperature range data were collected from type strain publications. (B) PC1 and PC2 of a PCoA of a binary matrix of gene presence/absence for 51 species of *Moraxellaceae*, explaining 32% and 14% of the variation, respectively. Each genome is represented by one point, colored by genus. The P-clade *Moraxella* spp. are represented by yellow points with red outlines. (C) PC2 and PC3, explaining 14% and 5% of the variation, respectively.

To explore the evolutionary history of *Psychrobacter*, we built a phylogeny based on 400 conserved marker genes using publicly available whole genomes derived from cultured isolates of 51 species from the *Moraxellaceae* family. We included 18 species each of Acinetobacter and *Moraxella* obtained from NCBI and 15 *Psychrobacter* genomes that we generated in this study (see Table S1 in the supplemental material). For all genomes, we incorporated phenotypic data collected from previously published type-strain research. The 51 species formed 3 distinct clades, each with robust bootstrap support. The Acinetobacter clade consists uniquely of Acinetobacter species (labeled A in Fig. 1A). The Acinetobacter clade is a sister taxon to the *Moraxella* (M) clade, consisting entirely of *Moraxella* species, and to the *Psychrobacter* (P) clade, which contains all of the *Psychrobacter* species, as well as 4 *Moraxella* species—M. boevrei, M. atlantae, M. osloensis, and M. lincolnii—that are basal.

10.1128/mSystems.00258-21.1 TABLE S1 *Moraxellaceae* genomics. Eighteen species each of Acinetobacter and *Moraxella* were chosen based on availability of information on isolation source and cultivation temperatures, and their genomes were downloaded from NCBI. Fifteen additional *Psychrobacter* accessions were chosen for the family-level analysis based on phylogenetic breadth. Download Table S1, XLSX file, 0.01 MB. Copyright © 2021 Welter et al. 2021 Welter et al. https://creativecommons.org/licenses/by/4.0/ This content is distributed under the terms of the Creative Commons Attribution 4.0 International license.

Consistent with the topology of the phylogeny, a principal-coordinate analysis (PCoA) of gene presence/absence data shows that the greatest variation within the family (i.e., principal coordinate [PC] 1) is the separation between the A clade and the M and P clades, which are grouped (Fig. 1). Genes annotated with very diverse functions, falling under almost every Cluster of Orthologous Groups (COG) category, strongly contribute to the separation between clades according to envfit analysis (Fig. 1B; Table S2). The P-clade *Moraxella* spp. fall between the P-clade *Psychrobacter* and the M-clade *Moraxella* when visualizing PC1 and -2 (Fig. 1B) as well as PC2 and -3 (Fig. 1C).

10.1128/mSystems.00258-21.2 TABLE S2 COG separation of PCoA. An envfit analysis was performed to identify functions that might be correlated with separation of accessions along a gene presence/absence principal-component analysis, for a PCoA at the *Moraxellaceae* family level as well as the *Psychrobacter* genus level. For every COG category, the *r*^2^, indicating the strength of the correlation; the *P* value, indicating the significance of the correlation; and the Benjamini-Hochberg-corrected *P* value are reported. Download Table S2, XLSX file, 0.01 MB. Copyright © 2021 Welter et al. 2021 Welter et al. https://creativecommons.org/licenses/by/4.0/ This content is distributed under the terms of the Creative Commons Attribution 4.0 International license.

Despite their close phylogenetic relationship and high similarity in gene presence/absence, *Psychrobacter* and *Moraxella* have different genomic properties. When examined by genus rather than clade, *Psychrobacter* species have an average genome size of 3.12 ± 0.27 Mb, while the average of *Moraxella* is 2.41 ± 0.28 Mb (pairwise Wilcoxon rank sum test [WRS], *P* value = 8e−07). *Moraxella* species have an average coding density of 86.4% ± 1.33%, which is significantly higher than the *Psychrobacter* species average of 82.7% ± 1.33% WRS, *P* value = 1e−5). Despite being phylogenetically more related to *Psychrobacter* species than to the other *Moraxella* species, the P-clade *Moraxella* strains are significantly different from *Psychrobacter* species for both genome size and coding density (pairwise WRS, *P* values = 0.004) while not significantly different from the M-clade *Moraxella* spp. (pairwise WRS, *P* values = 0.9).

### *Psychrobacter* ranges of growth temperatures differ from those of *Moraxella*.

**FIG 2:**
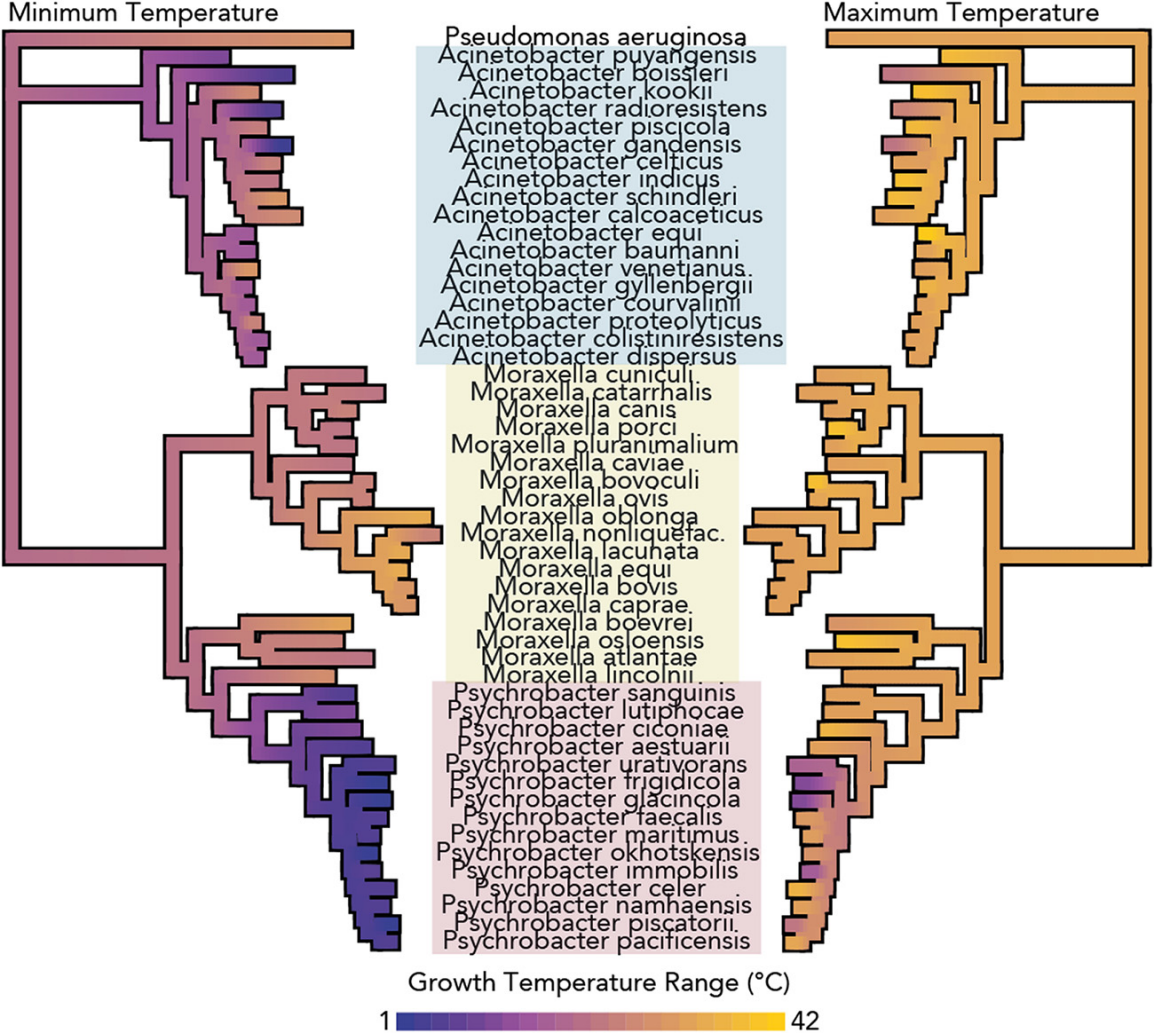
Psychrophily is observed only in *Psychrobacter* spp. among the family *Moraxellaceae*. Continuous trait mapping growth temperature ranges of 51 species from the *Moraxellaceae* family, taken from type strain publications. Values at nodes are imputed by maximum likelihood analysis. The phylogeny was constructed by marker-gene analysis including 400 genes, as in Fig. 1. Genera are indicated with colored boxes.

To examine the phenotypic behaviors of the *Moraxellaceae* family, we applied continuous trait mapping of the ranges of temperatures at which species from the *Moraxellaceae* family can grow (16, 17, 20, 21, 26, 28–68) onto the previously generated phylogeny (Fig. 2). The *Psychrobacter* spp. included here exhibit a broad range of growth temperatures (0 to 38°C), but several strains, such as P. frigidicola and P. glacincola, are psychrophilic (restricted to growth below 20°C), which is a phenotype that is not seen elsewhere in the family. *Psychrobacter* spp. have lower minimum growth temperatures than the included *Moraxella* spp. from either the P or M clades (pairwise WRS, *P* value = 5e−06), which have a narrow range of temperatures at which they can grow (between 22°C and 40°C). In contrast to the minimum growth temperatures of the family, there is little variation in the maximum growth temperatures, except for the notable exceptions of several *Psychrobacter* spp. that are entirely restricted to low temperatures.

As with their genomic properties, P-clade *Moraxella* strains have more similar phenotypes compared to M-clade *Moraxella* strains than to *Psychrobacter* strains. Review of type strain descriptions revealed that *Psychrobacter* strains are consistently urease positive, nitrate reducing, salt tolerant, and nonfastidious, whereas P-clade *Moraxella* strains are inconsistent with their urease- and nitrate-reducing phenotypes, are sensitive to high salt concentrations, and are often nutritionally fastidious—they have complex growth requirements (in particular, often blood or bile for growth) (16, 17, 26, 32, 35, 43, 44, 59).

### Few *Psychrobacter* strains can grow at 37°C.

To explore *Psychrobacter* phenotypic diversity, we established a strain collection of 85 *Psychrobacter* accessions isolated from diverse locations and ecological sources (Table S3). To characterize *Psychrobacter* phenotypes under a number of different conditions, we assessed the strain collection for resistance to bile salts as well as ability to grow under 24 different conditions: two different media combined with four different salt concentrations and with three different temperatures (Materials and Methods).

10.1128/mSystems.00258-21.3 TABLE S3 *Psychrobacter* cultivation. Summary of information from strain catalogues about the cultivation media and temperatures, isolation information, previously published genomes, and phenotyping batch of each of the 92 *Psychrobacter* strains initially included in our strain collection, with notes about the 7 strains that were ultimately not used for the analysis. Download Table S3, XLSX file, 0.02 MB. Copyright © 2021 Welter et al. 2021 Welter et al. https://creativecommons.org/licenses/by/4.0/ This content is distributed under the terms of the Creative Commons Attribution 4.0 International license.

**FIG 3:**
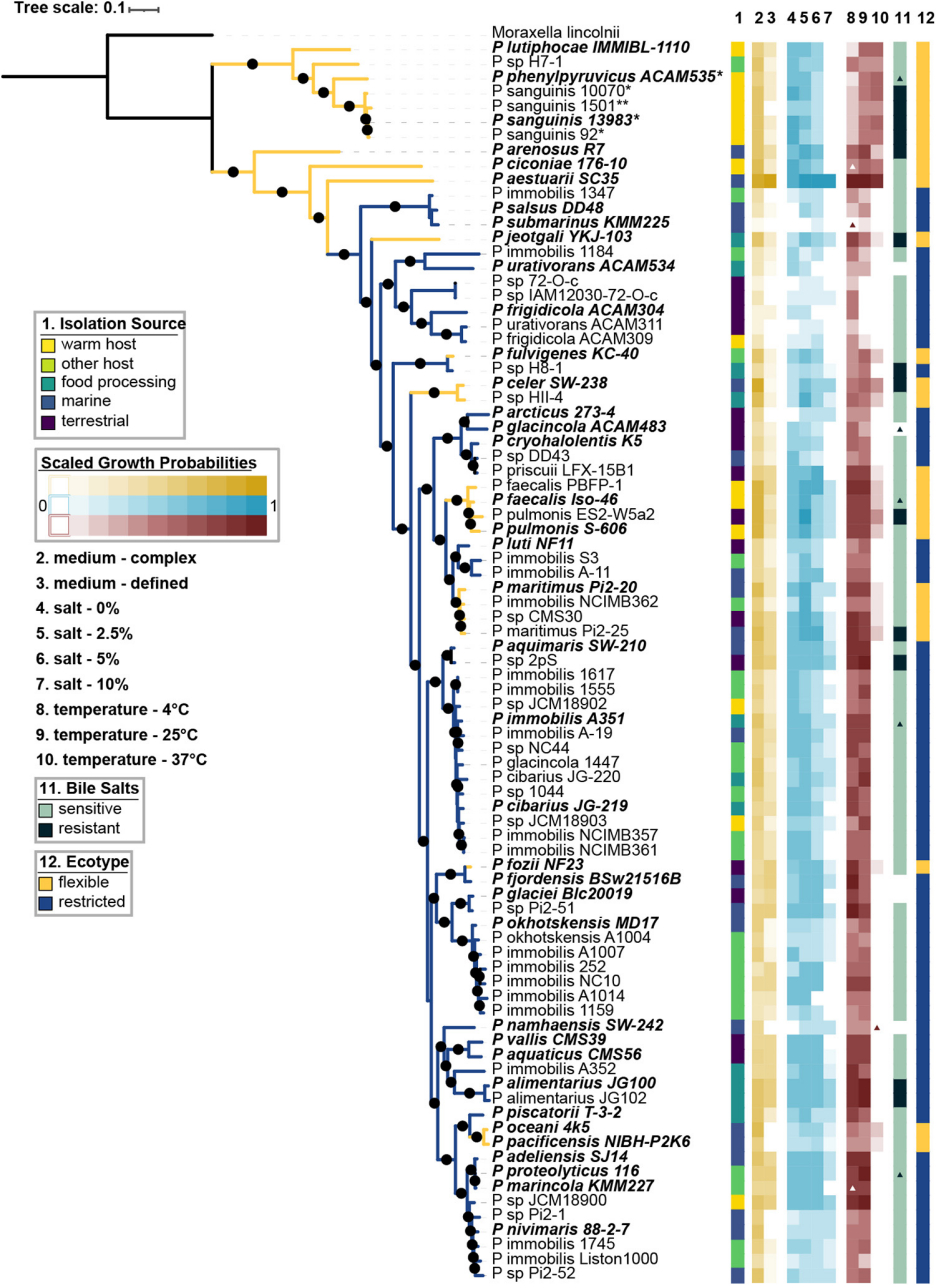
*Psychrobacter* phenotypic and phylogenomic diversity. The *Psychrobacter* phylogeny was constructed using FastTree with 1,000 bootstraps, refined by RAxML under the PROTCATG model. Amino acid sequences from 400 marker genes were used in the alignment. Branches with bootstrap support greater than 70% are represented by filled circles. The scale bar represents the average amino acid substitutions per site. *M. lincolnii* was used as an outgroup. Type strain isolate names are indicated in bold and italicized type. Strains indicated with * next to their name exhibited growth defects in liquid media and were tested on solid agar media instead. Strains indicated with ** exhibited growth defects on solid and liquid media and were tested on solid media supplemented with 0.1% Tween 80. Isolation source is depicted in column 1 as a color strip. Columns 2 to 10 represent the growth probabilities of each strain for each condition; medium complexity is represented in yellow, salt concentration is represented in blue, and temperature is represented in red. Type strain data support our temperature data except where indicated—colored triangles show conditions under which we expected growth but did not observe it, while white triangles represent conditions under which we observed growth that we did not expect. Bile salt resistance is shown in column 11, with black triangles indicating strains where we expected resistance but observed sensitivity, and green triangles representing the opposite. The “ecotype” is shown in column 12 in a color strip and in the color of the branches.

We calculated growth probabilities, or the fraction of growth-positive conditions out of total conditions tested, for every strain and given variable of the growth curve screen and compared them along with the bile resistance data across the phylogeny and by isolation source (Fig. 3). We generated a robust genus-level phylogeny for *Psychrobacter* using 400 conserved marker genes, with *M. lincolnii* as an outgroup. In agreement with single marker gene trees generated using *rpoB* sequences (27) and 16S rRNA gene sequences (69) as well as the P-clade structure of the *Moraxellaceae* family tree generated in this study, we observed a phylogenetically basal group of strains mostly isolated from mammals, and a phylogenetically derived group isolated from mixed sources. Across the entire phylogeny, closely related strains have similar growth probabilities (Pagel’s λ ranging from 0.78 to 0.97, all corrected *P* values < 1e−3). We observed that most *Psychrobacter* strains are tolerant of a wide variety of temperatures between 4 and 25°C and of salt concentrations between 0 and 5%; more than 90% of all strains can grow under these conditions. However, only 54% of the tested accessions can grow at 10% added salt, and only 31% can grow at 37°C.

Since we were interested in the possibility of *Psychrobacter* interacting with warm-bodied hosts, we divided the strains into two ecotypes: the “flexible ecotype” (FE) corresponds to strains that could grow at 37°C, and the “restricted ecotype” (RE) corresponds to strains that could not grow at 37°C. FE strains are psychrotrophic (mesophilic organisms with a low minimum growth temperature but an optimal growth temperature above 15°C), and RE strains are either psychrotrophs or true psychrophiles (unable to grow at temperatures higher than 20°C).

Notably, the basal clade of the *Psychrobacter*-only tree (Fig. 3) consists solely of FE strains, while the rest of the phylogeny is made up both FE and RE strains. Furthermore, the basal FE strains have higher growth probabilities at 37°C than do other FE strains (Pagel’s λ = 0.89, *P* value = 2e−5). Frequencies of RE and FE strains vary significantly across sources of isolation: the FE group is significantly enriched in strains derived from mammalian sources, and the RE group is significantly enriched in strains derived from other hosts, food, and marine and terrestrial sources (χ^2^ test, 83% of *P* values adjusted for group size < 0.05). Nonetheless, both FE and RE contain strains from other environments, including mammalian-derived strains within the RE group.

FE strains show higher growth probabilities in complex media than in defined media, while RE strains showed little difference between the two (WRS, Wilcoxon test statistic [W] = 1.13e3, *P* value = 0.0005, phylogenetic *P* value = 0.002). FE strains also have higher growth probabilities at low to medium salt concentrations (WRS, all *P* values < 0.05, phylogenetic *P* values < 0.4), though there is no difference between FE and RE strains at 10% salt (WRS, W = 811.5, *P* value = 0.7, phylogenetic *P* value < 0.4). Finally, FE strains are more likely to be resistant to bile salts than RE strains (χ^2^ test, 93% of *P* values adjusted for group size < 0.05).

### FE and RE *Psychrobacter* spp. have functional and structural genomic differences.

**FIG 4:**
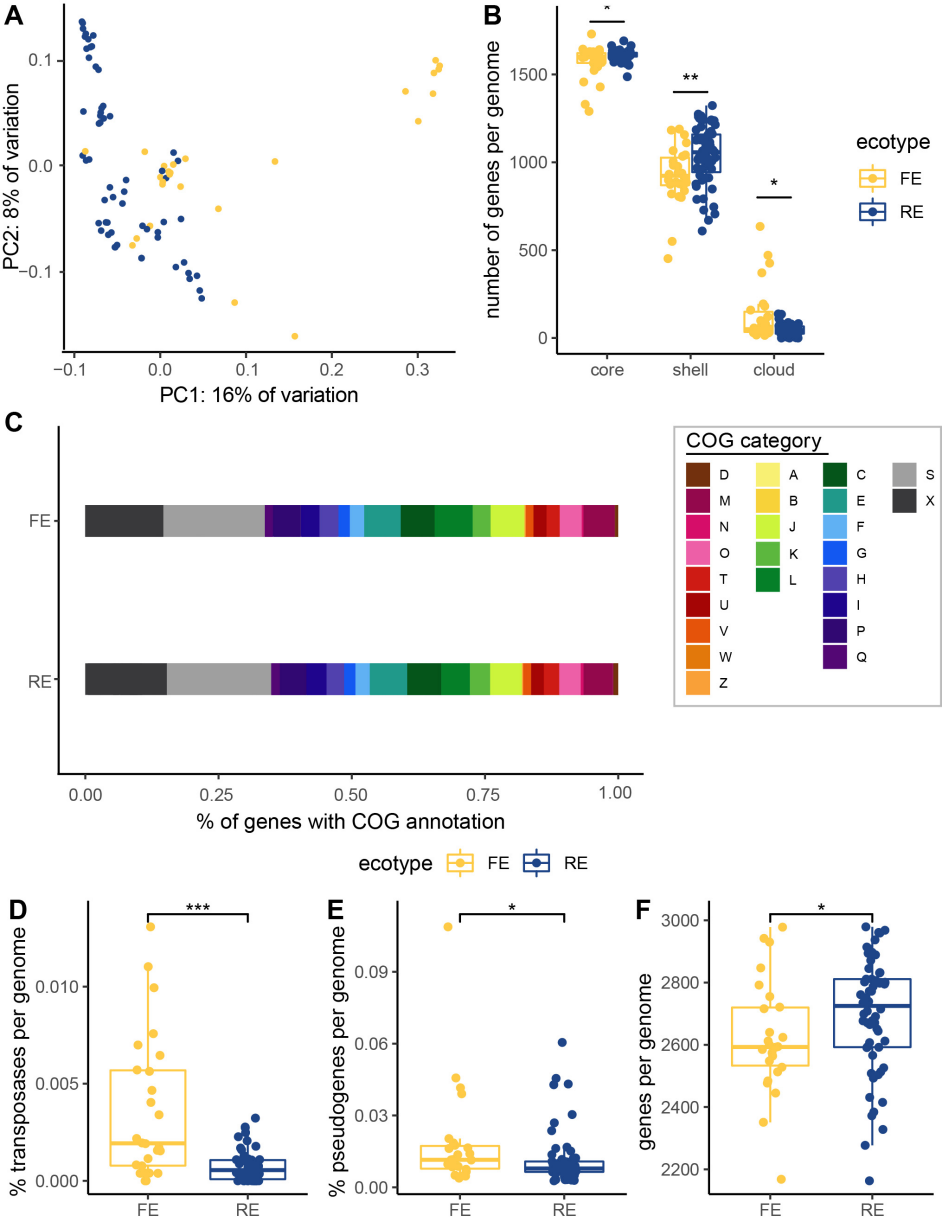
Structural differences between the genomes of FE and RE *Psychrobacter* strains. (A) The first two axes of a PCoA of a gene presence-absence matrix of all of the included accessions, colored by ecotype. (B) Pangenome categories. (C) Proportions of the average genome of each ecotype devoted to each cluster of orthologous genes (COG) category. Cellular processing and signaling: [D] cell cycle control cell division, chromosome partitioning; [M] cell wall/membrane/envelope biogenesis; [N] cell motility; [O] posttranslational modification, protein turnover, and chaperones; [T] signal transduction mechanisms; [U] intracellular trafficking, secretion, and vesicular transport; [V] defense mechanisms; [W] extracellular structures; [Y] nuclear structure; [Z] cytoskeleton. Information storage and processing: [A] RNA processing and modification; [B] chromatin structure and dynamics; [J] translation, ribosomal structure and biogenesis; [K] transcription; [L] replication, recombination, and repair. Metabolism: [C] energy production and conversion; [E] amino acid transport and metabolism; [F] nucleotide transport and metabolism; [G] carbohydrate transport and metabolism; [H] coenzyme transport and metabolism; [I] lipid transport and metabolism; [P] inorganic ion transport and metabolism; [Q] secondary metabolites biosynthesis, transport, and catabolism. Poorly characterized: [S] function unknown, [X] not in COG database. (D) Percent of genes that are transposases. (E) Percent of genes per genome that are predicted to be pseudogenes. (F) Number of genes per genome. Means were compared using the Wilcoxon rank sum test. *, *P* value less than 0.05; **, *P* < 0.005; ***, *P* < 0.0005.

When exploring gene presence/absence via PCoA, accessions from the basal FE-only subclade cluster closely together, indicating similar gene content. In agreement with the phylogeny, these basal FE accessions are separated from the other accessions on PC1, while the derived FE and RE accessions are more scattered, indicating more diverse gene content (Fig. 4A). According to an envfit analysis, the separation is most strongly driven by genes from the COG categories T, signal transduction (*r*^2^ = 0.65, *P* value = 0.001); U, trafficking and secretion (*r*^2^ = 0.63, *P* value = 0.001); P, inorganic ion transport and metabolism (*r*^2^ = 0.56, *P* value = 0.001), and X, unassigned or no homologs in the COG database (*r*^2^ = 0.51, *P* value = 0.001) (Table S2).

The average *Psychrobacter* genome carries 2,763 genes, 1,598 of which are core (present in 90 to 100% of strains), 1,005 are shell (present in 2 or more strains but fewer than 90%), and 70 are cloud (present in only one strain). The average FE genome has significantly fewer core (WRS, W = 531.5, *P* value = 0.02, phylogenetic *P* value = 0.3) and shell (WRS, W = 462, *P* value = 0.003, phylogenetic *P* value = 0.4) genes but significantly more cloud genes (WRS, W = 1014, *P* value = 0.02, phylogenetic *P* value = 0.2) than the average RE genome (Fig. 4B).

In terms of functional differentiation between the ecotypes, RE strains’ genomes have a higher proportion of “W” (extracellular structure) genes (WRS, W = 275, adjusted *P* value = 0.00006, phylogenetic *P* value = 0.04), “I” (lipid transport and metabolism) genes (WRS, W = 437, adjusted *P* value = 0.01, phylogenetic *P* value = 0.2), and “S” (function unknown) genes (WRS, W = 492, adjusted *P* value = 0.04, phylogenetic *P* value = 0.4). FE strains’ genomes have a higher proportion of “P” (inorganic ion transport and metabolism) genes (WRS, W = 1.09e3, adjusted *P* value = 0.01, phylogenetic *P* value = 0.8). They also have a significantly higher proportion of COG category “L” (replication-, recombination-, and repair-related) genes (WRS, W = 1.10e3, adjusted *P* value = 0.01, phylogenetic *P* value = 0.3) (Fig. 4C) and, in particular, have higher copy numbers of transposons (WRS, W = 1.08e3, *P* value = 0.003, phylogenetic *P* value = 0.1) than do RE strains’ genomes (Fig. 4C and E).

Increased transposon activity can lead to interruption and decay of functional protein-encoding genes, leading to an increase in pseudogenes (70). Given the higher number of transposons in FE strains, we next examined the number of predicted pseudogenes between the ecotypes. FE genomes are predicted to have a higher proportion of pseudogenes than RE strains (WRS, W = 997, *P* value = 0.03, phylogenetic *P* value = 0.6) (Fig. 4C). Finally, we compared the average numbers of genes per genome between the ecotypes, as bacterial genomes are known to strongly select against the accumulation of pseudogenes resulting in gene deletions (71), and found that FE strains have significantly fewer genes than RE strains (WRS, W = 547, *P* value = 0.05, phylogenetic *P* value = 0.6).

We looked for specific gene clusters differentiating the FE and RE ecotypes while controlling for population structure by performing a microbial pangenome-wide association analysis with the R package treeWAS (72). This returned four gene clusters that significantly correlated with ecotype, each of which is enriched in RE strains compared to FE strains (Table 1). The gene with the most significant enrichment is annotated as a transcriptional regulator in the TetR/AcrR family with the KEGG KOs of K16137 and K19335, which is predicted to be either *nemR* or *bdcR*. Other significant results include *lpdA*, dihydrolipoamine dehydrogenase related to amino acid metabolism (KEGG KO K00382/383), a hypothetical protein with the IG domain, and a hypothetical protein with no homologs in eggNOG database and no predicted annotation. Each of these genes has paralogs that are not significantly enriched in one ecotype over the other.

**TABLE 1:** Gene clusters detected as significantly associated with *Psychrobacter* ecotype based on a pangenome-wide association analysis^*a*^

Gene cluster	Paralogs^*b*^	Predicted gene name	KEGG KO	COG category	eggNOG annotation	No. of FE strains	No. of RE strains	% FE strains	% RE strains
GC00000020	GC00000020_1	*merA*	K00382, K00383, K00520	C	Mercuric reductase	0	1	0	2
GC00000020	GC00000020_2	*merA*	K00382, K00383, K00520	C	Mercuric reductase	4	4	15	7
GC00000020	GC00000020_3	*lpd*	K00382	C	Dihydrolipoamide dehydrogenase	2	1	8	2
GC00000020	GC00000020_4	*lpd*	K00382	C	Dihydrolipoamide dehydrogenase	1	0	4	0
GC00000020	GC00000020_5	*lpd*	K00382	C	Dihydrolipoamide dehydrogenase	25	59	96	100
GC00000020	GC00000020_6***	*lpdA*	K00382, K00383	C	Dihydrolipoamide dehydrogenase	9	53	35	90
GC00000020	GC00000020_7		K00383	C	Pyridine nucleotide-disulfide oxidoreductase	0	3	0	5
GC00000020	GC00000020_8	*sthA*	K00322, K00382, K17883	C	Conversion of NADPH to NADH	26	59	100	100
GC00000020	GC00000020_9	*gor*	K00383	O	Glutathione reductase	23	46	88	78
GC00000020	GC00000020_10	*lpdG*	K00382	C	Dihydrolipoyl dehydrogenase	26	59	100	100
GC00001343	GC00001343_1		K16137	K	TetR family transcriptional regulator	0	1	0	2
GC00001343	GC00001343_2		K16137	K	Transcriptional regulator	0	1	0	2
GC00001343	GC00001343_3		K16137	K	TetR family transcriptional regulator	3	0	12	0
GC00001343	GC00001343_4		K16137, K19335	K	Transcriptional regulator TetR family	1	0	4	0
GC00001343	GC00001343_5		K16137, K19335	K	Transcriptional regulator TetR family	5	16	19	27
GC00001343	GC00001343_6***		K16137, K19335	K	Transcriptional regulator TetR family	5	52	19	88
GC00001837	GC00001837_1***			S	Ig domain protein group 1 domain protein	1	11	4	19
GC00001837	GC00001837_r1_1			S	Ig domain protein group 1 domain protein	1	0	4	0
GC00001837	GC00001837_r1_r1_1			S	Ig domain protein group 1 domain protein	12	26	46	44
GC00001839	GC00001839_1			X		1	0	4	0
GC00001839	GC00001839_r1_1***			X		6	47	23	80

a Gene cluster refers to a group of protein-coding gene sequences with distant sequence homology which may be either orthologs or paralogs, while paralog refers to subsets within the gene cluster identified as exact gene matches.

b ***, the paralogs identified as significantly associated with ecotype by the pangenome-wide association software treeWAS.

### Polar bear feces collected from the Arctic ice have a high abundance of *Psychrobacter*.

**FIG 5:**
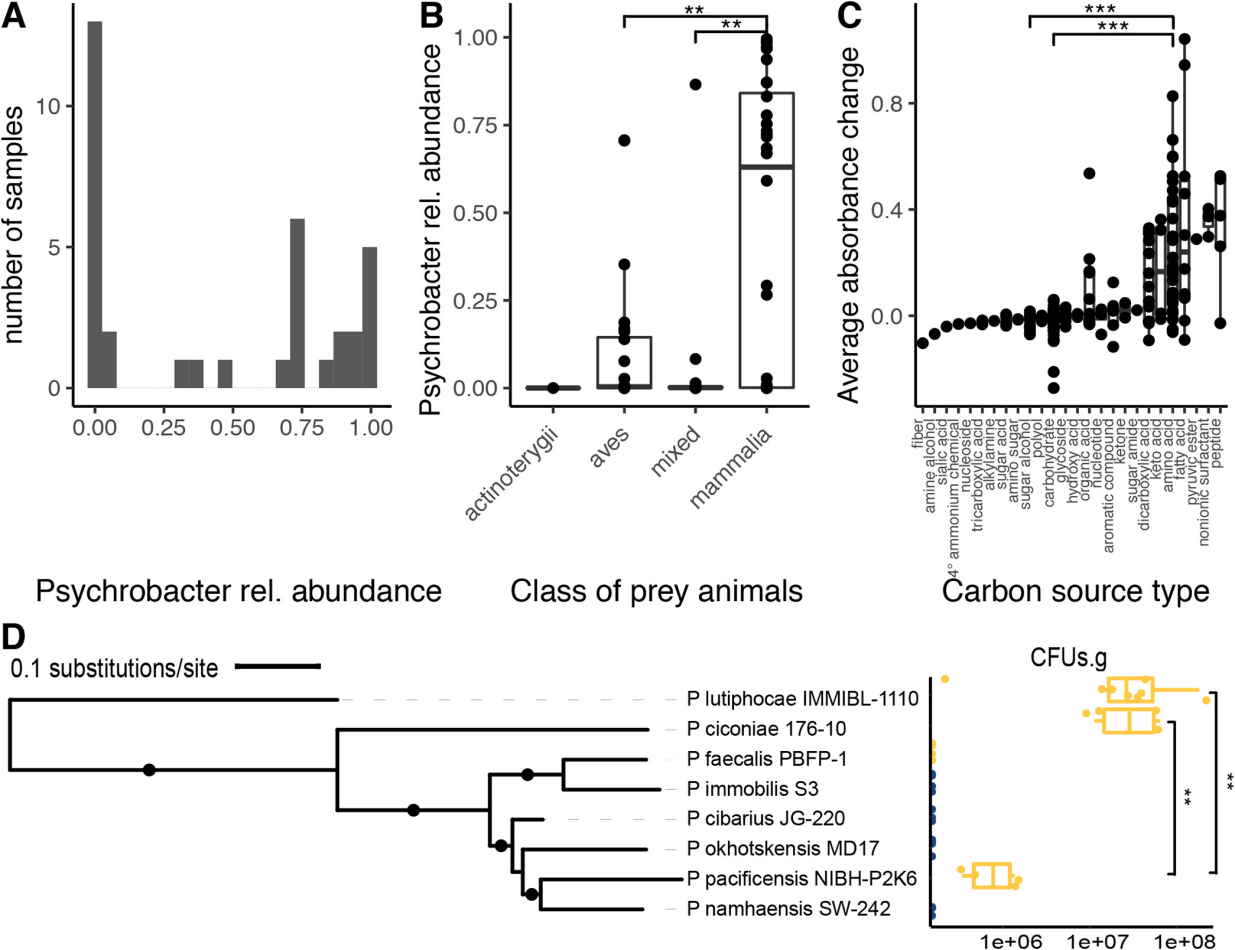
*Psychrobacter* strains occur and persist in the mammalian gut. (A) Histogram of relative abundance at the genus level of 16S rRNA gene sequence variants (SVs), clustered at 100% identity, of polar bear fecal samples. (B) *Psychrobacter* relative abundance in comparison to taxonomy of prey consumed by polar bears. (C) The average change in OD_600_ of 19 *Psychrobacter* accessions grown on 190 different substrates as sole carbon sources, compared across different classes of compounds. (D) CFU per gram of cecal contents of gnotobiotic mice is shown in comparison to accession phylogeny. FE strains are shown in yellow, while RE strains are shown in blue. The phylogeny was constructed as described in the legend to Fig. 3. Branches with bootstrap support higher than 70% are indicated with a filled circle. Means were compared using the Wilcoxon rank sum test. *, *P* value less than 0.05; **, *P* < 0.005; ***, *P* < 0.0005.

*Psychrobacter* spp. have been reported previously in the skin, respiratory, and gut microbiomes of several marine mammals, including whales, porpoises, seals, and sea lions; *Psychrobacter* presence may stem from the surrounding seawater. Polar bears, on the other hand, are marine mammals that do not spend as much time swimming. We surveyed the gut microbial diversity of 86 polar bear fecal samples, 76 wild and 10 captive, by 16S rRNA gene sequencing. Sequence variant clustering of the 16S rRNA gene sequences reveals that *Psychrobacter* is detectable in 88% of the samples (Fig. 5A). The large majority of *Psychrobacter* sequences were assigned to unclassified *Psychrobacter* spp., which are prevalent in 87% of samples with a mean abundance of 22%. We also detected RE strain P. immobilis in 49% of samples with a mean abundance of 3%, and FE strain P. pulmonis in 8% of samples with a mean abundance of 0.5%. Polar bear diet significantly impacts the abundance of *Psychrobacter* spp. (Kruskal-Wallis χ^2^ = 13.5, df = 3, *P* value = 0.004); we found that polar bears feeding on mammalian prey, including seals and reindeer, had significantly higher abundances of unclassified *Psychrobacter* spp. than polar bears feeding on avian prey or mixed diets (pairwise Wilcoxon rank sum test, adjusted *P* values < 0.05) (Fig. 5B). However, diet data are confounded with other metadata, as location (Kruskal-Wallis χ^2^ = 8.6, df = 4, *P* value = 0.0009) and year (Kruskal-Wallis χ^2^ = 16.6, df = 5, *P* value = 0.005) of sample collection also significantly impact unclassified *Psychrobacter* abundance. Captive status does not significantly impact abundance, but there is a trend of wild bear samples having higher unclassified *Psychrobacter* abundance than samples from captive bears (WRS, W = 222, *P* value = 0.08).

To elucidate the effect that host dietary nutrition may have on *Psychrobacter* growth, we tested the maximum change in absorbance for 190 different carbon sources by a subset of *Psychrobacter* accessions including 9 FE and 10 RE strains (Fig. 5C). All *Psychrobacter* spp. reached significantly higher optical densities at 600 nm (OD_600_) growing on amino acid carbon sources compared to carbohydrates or sugar alcohols (pairwise WRS, adjusted *P* values < 0.05). *Psychrobacter* spp. reached the highest OD_600_ growing on fatty acids, surfactants, and peptides, but there was no significant difference between FE and RE strains’ changes in absorbance (WRS, W = 1.55e6, *P* value = 0.08), though there is a slight trend of RE strains having overall higher changes in absorbance.

### *Psychrobacter* strain survival in gnotobiotic mice.

To assess the survivorship of *Psychrobacter* in a mammalian gut, we tested 8 accessions for persistence in the gastrointestinal tracts of germfree mice (Fig. 5D). Choosing for phylogenetic breadth, we tested 4 FE strains (P. ciconiae, P. faecalis PBFP-1, P. lutiphocae, and P. pacificensis) and 4 RE strains (P. cibarius JG-220, *P. immobilis* S3, P. namhaensis, and P. okhotskensis MD17). Of the four FE strains tested, three were able to persist in the mice, while the FE strain *P. faecalis* PBFP-1 and none of the RE strains were detectable after 3 weeks. Phylogenetic relatedness does not correlate with ability to colonize, as FE strain *P. pacificensis* was able to persist, while closely related RE strains *P. namhaensis* and *P. okhotskensis* were not. Phylogenetic placement does correlate with colonization density, however, as the two most basal strains tested, *P. lutiphocae* and *P. ciconiae*, colonize at significantly higher densities than the most derived strain that was successful, *P. pacificensis* (pairwise WRS, both adjusted *P* values = 0.0007).

## DISCUSSION

The phylogenomic and phenotypic characterization of the *Psychrobacter* genus indicates a common ancestor with *Moraxella*, all of which are restricted to growth at higher temperatures. Furthermore, the most basal members of the *Psychrobacter* clade are *Moraxella* species and species of *Psychrobacter* that can grow at 37°C, unlike most of the derived *Psychrobacter* species. Our extensive phenotyping indicated that members of the *Psychrobacter* genus grow at a wide range of salinities and temperatures, but distinguishing strains by their ability to grow at 37°C results in two groups of strains with differences in response to rich versus defined media, bile salt resistance, and genomic characteristics. Our analysis of a collection of wild polar bear species indicates both RE and FE strains are present; however, tests in germfree mice support the notion that only FE strains can colonize the mammal gut, whereas RE strains may be allochthonous members. Together with previous reports, this work indicates the genus *Psychrobacter* is a lineage descended from pathobionts, some of which have evolved to inhabit the colder environments of their warm-bodied hosts.

Our results corroborate those of Bakermans, who used the isolation source of *Psychrobacter* as a proxy for temperature adaptation to conclude the genus has a mesophilic ancestor (27). By assessing growth under the same 24 conditions for 85 strains, we remove any ambiguity that can stem from whether an isolate can indeed grow at the temperature of its source of isolation. This is particularly important given that several strains isolated from mammals proved to be RE and that many of the FE strains came from seawater or other relatively cold environments. We also expanded upon the results of Bakermans by including phylogenetically aware statistics. In any population, identifying traits that are shared due to ecological convergence versus those that are shared due to common ancestry is a well-recognized problem (73); this problem is particularly striking in the genus *Psychrobacter*, where not only is there phenotypic divergence between the more phylogenetically basal strains and the derived strains, but as evidenced by the gene presence/absence PCoA, there is a large difference in gene content based on phylogeny.

We found that a transcriptional repressor, either NemR or BdcR, is enriched in RE strains’ genomes compared to FE strains. NemR plays a role in electrophile sensing (74), while BdcR plays a role in cyclic-di-GMP sensing (75). Both relate to increased biofilm formation when expressed. Similarly, we found a predicted Ig-domain protein, which is thought to be an outer membrane protein with a role in adhesion (76), enriched in RE strains. Adhesion and biofilm formation can increase during cold stress (77), indicating that it may be adaptive for psychrophiles or psychrotrophs to have increased biofilm formation capability.

In addition to specific genes being enriched in RE strains, we found broader functional differences between the ecotypes. FE strains had higher proportions of COG category “L” (replication) and “P” (inorganic transport and metabolism) genes. The higher proportion of “P” category genes could point to FE strains’ association with hosts, as free iron availability is rather low within host bodies (78). The higher “L” proportion seems to be related to higher transposon copy numbers in FE strains, which could point to population bottlenecks relating to host association (79). RE strains, on the other hand, showed increased proportions of COG category “W” (extracellular structures), “S” (function unknown), and “I” (lipid metabolism) genes. The increase in the proportion of “W” genes is unlikely to have a large impact on phenotypic differences between the ecotypes, since there are very few “W” genes in genomes of either RE or FE strains. It is difficult to comment on the increased proportion of “S” genes, since their function is unknown or poorly understood. The increased proportion of “I” category genes, relating to lipid transport and metabolism, could point to increased flexibility in membrane lipids for RE strains, which is a well-characterized adaptation to cold temperatures (80).

The three of four FE strains tested were able to colonize germfree mice, whereas the RE strains could not, indicating that growth at 37°C may be necessary (although not sufficient) to colonize mammals. Opportunistic infections in mammals caused by *Psychrobacter* strains are limited to P. sanguinis, P. phenylpyruvicus, *P. faecalis*, and *P. pulmonis* (25), which are all FE strains but do not all belong to the basal clade. Our results suggest that the FE strains maintain an ancestral ability to grow at mammalian body temperatures and colonize mammalian host bodies, while RE strains have adapted toward psychrophilic or psychrotrophic lifestyles.

*Psychrobacter*’s sister taxon *Moraxella* in particular is commonly isolated from host mucosal tissues and exhibits the reduced genome size and nutritional fastidiousness common to many host-dependent organisms. *Moraxella* contains species that are frequently associated with human respiratory infections, primarily M. catarrhalis (81), as well as livestock conjunctivitis, for example, M. bovis or M. equi (36). Since they are commonly found in healthy individuals and can cause disease in healthy individuals (82), *Moraxella* bacteria are best categorized as pathobionts and not dedicated or opportunistic pathogens. Several species of *Moraxella* appear basally in the P clade of the *Moraxellaceae* family-level phylogeny, suggesting that *Psychrobacter* evolved from a “*Moraxella*-like” ancestor. This is supported by the fact that both phylogenetically basal and derived *Psychrobacter* strains carry genes related to virulence functions and that many of the basal *Psychrobacter* strains exhibit growth defects in liquid culture, similar to the fastidiousness of *Moraxella*.

Despite clear phenotypic differentiation, *Psychrobacter* and *Moraxella* have similar genomic content, although *Psychrobacter* genomes are larger. A psychrophile emerging from an apparently mesophilic background through widespread horizontal gene transfer has been suggested before in the genus *Psychroflexus* (83), though the study was limited to comparing only two genomes. In fact, it has been suggested before that this is *Psychrobacter*’s evolutionary trajectory (27), and although many *Psychrobacter* trees are constructed using a *Moraxella* outgroup, *Psychrobacter*’s potential pathobiont origin has not been explored in detail. Horizontal gene transfer would explain *Psychrobacter*’s larger genome size compared to *Moraxella* despite lower coding density, as many newly acquired horizontally transferred genes are expected to be inactivated and pruned by the recipient genome (84, 85).

Our results indicate that the history of the genus *Psychrobacter* is that of an ancestral pathobiont or pathogen, some of the descendants of which broadened their ecological distribution, resulting in an attenuation of pathogenicity. The emergence of a psychrotroph—a remarkable generalist—from a background of a more specialized pathobiont or pathogen showcases the remarkable adaptability of bacteria, and particularly *Proteobacteria*, to their environments.

## MATERIALS AND METHODS

### *Moraxellaceae* family genomics and phenotypic data.

The *Moraxellaceae* family includes 3 well-characterized genera: *Moraxella*, Acinetobacter, and *Psychrobacter*. To build a family-level phylogeny, we downloaded genomes of 18 species of Acinetobacter and 18 species of *Moraxella* from the National Center for Biotechnology Information (NCBI) genome database (86) (June 2020) (see Table S2 in the supplemental material). We also included 15 *Psychrobacter* genomes generated in this study (described below). We determined the phylogenetic relationship between genomes using the whole-genome marker gene analysis software PhyloPhlAn (87) (v2.0, diversity = “medium.” set to “accurate”) and determined genome quality and summary characteristics using CheckM (88) (v1.0.18) and Prokka (89) (v1.14.6, kingdom = “bacteria”). The *Moraxellaceae* phylogenetic tree was visualized and annotated using the interactive Tree of Life (iTOL) web interface (90) (v5.6.1).

We analyzed the *Moraxellaceae* pangenome using the PanX pipeline (91) (v1.6.0, core gene cutoff = 0.9). The input genomes used by PanX were initially annotated using Prokka. After their assignment into orthologous clusters using MCL (v14.137), we reannotated gene clusters using eggNOG-Mapper (92) (v1.0.3). We explored genome content by calculating a distance matrix using the Jaccard metric through the R package ecodist (93) (v0.3.0) from a binary gene presence-absence table, followed by dimensional reduction of that distance matrix through principal-coordinate decomposition (PCoA) with the cmdscale function from the R package stats. We investigated variables contributing to the separation of the PCoA using the envfit function from the R package vegan (94) (v2.5-6); we tested whether genes significantly contributing to separation were “core” (genes present in 90% to 100% of strains), “shell” (genes present in greater than two strains, but in fewer than 90%), or “cloud” (genes present in only one strain) and if general gene function—summarized by Cluster of Orthologous Groups (COG) category (95)—contributed to the separation. We collected growth temperature range data from type strain publications (16, 17, 20, 21, 26, 28–68). We used the R package phytools (96) (v0.7-47) to map the temperature ranges onto the phylogeny.

### *Psychrobacter* strains.

We obtained 92 isolates of *Psychrobacter* from strain catalogues for phenotypic and genotypic characterization. These represent 38 validly published species of *Psychrobacter* as well as unclassified strains, all isolated from a wide variety of geographical locations and diverse environmental and host samples. All strains were purchased and maintained in compliance with the Nagoya Protocol on Access to Genetic Resources and the Fair and Equitable Sharing of Benefits Arising from their Utilization to the Convention on Biological Diversity. For a full list of accessions and their catalogue, isolation, and cultivation information, see Table S3. Unless mentioned, we grew accessions as recommended by the strain catalogue (medium and temperature) from which they were purchased.

### *Psychrobacter* carbon utilization assay.

To inform the design of the minimal medium used in the phenotypic screen described below, we chose a subset of 19 *Psychrobacter* strains (P. adeliensis, P. aestuarii, P. aquaticus, P. arenosus, P. celer, *P. cibarius* JG-219, *P. ciconiae*, P. cryohalolentis, *P. faecalis* PBFP-1, P. fozii, P. fulvigenes, *P. glacincola* ACAM483, P. luti, *P. lutiphocae*, P. maritimus Pi2-25, *P. okhotskensi*s MD17, *P. pacificensis*, P. piscatorii, and P. urativorans ACAM534) to evaluate for their ability to grow on 190 different substrates as their sole carbon source. We utilized Biolog plates PM1 and PM2 (Biolog Incorporated, Hayward, CA, USA) following a slightly modified protocol.

Briefly, we streaked out strains on agar plates of their preferred medium incubated at their preferred temperature (Table S3). We scraped cells from these plates and resuspended them at a final optical density at 600 nm (OD_600_) of 0.07 in the Biolog inoculation fluid (PM IF-0a GN/GP, 1.2×) diluted to 1× with sterile water. We inoculated 100 μl of cell suspension into each well of the PM1 and PM2 plates, mixing well to resuspend the carbon sources. All strains were grown under aerobic conditions at their preferred growth temperature (Table S3). Growth was monitored by measuring OD_600_ for 14 days. We calculated total change in absorbance by subtracting the blank wells from the substrate wells and then taking the difference between the absorbances at *t* = 14 days and *t* = 0 (inoculation time point). We then averaged the total change in absorbance for all strains for every carbon source and assigned each carbon source to a “family” of compounds.

All strains reached a significantly higher max OD_600_ in amino acid substrates than in other carbon sources. l-Glutamate was chosen as the carbon source for the defined medium described in our phenotypic screens below, as none of the strains tested failed to grow using it. Some of the other amino acids allowed strains to grow to a larger change in OD_600_ but failed to allow all strains to grow.

### *Psychrobacter* phenotypic screen.

For the 85 *Psychrobacter* accessions that passed genome quality control (QC) (see Table S4), we tested their ability to grow under 24 different conditions, a growth condition being a combination of a medium (complex or defined), salt concentration (0, 2.5, 5, or 10% NaCl) and incubation temperature (4, 25, or 37°C).

10.1128/mSystems.00258-21.4 TABLE S4 Sequencing summary. Summary of the total number of short and long reads, as well as quality control information provided by CheckM, such as completeness, contamination, genome size, and scaffold information, for all 92 *Psychrobacter* accessions included in our initial strain collection. Some strains were removed from further analysis due to poor-quality genomes. Download Table S4, XLSX file, 0.02 MB. Copyright © 2021 Welter et al. 2021 Welter et al. https://creativecommons.org/licenses/by/4.0/ This content is distributed under the terms of the Creative Commons Attribution 4.0 International license.

We used lysogeny broth (LB) as a complex medium with high nutrient availability and a variation of M9 minimal medium (MM) as a defined medium with lower nutrient availability. We prepared LB following the manufacturer’s instructions except by various concentrations of sodium chloride (NaCl) (see below) and autoclaving it at 121°C for 20 min to ensure sterility. We prepared MM by adding components in the following final concentrations, followed by filter sterilization: 33.7 mM Na_2_HPO_4_, 22.0 mM KH_2_PO_4_, 9.35 mM NH_4_Cl, 0.4% l-glutamic acid, 1 mM MgSO_4_, 0.3 mM CaCl_2_, 1× ATCC trace vitamins solution, and 1× ATCC trace minerals solution, pH 7 (adjusted with KOH). We added NaCl to each “base medium” to 1 of the 4 following concentrations of NaCl: low, with 0%; medium, with 2.5%; medium-high, with 5%; and high, with 10%. We grew cultures at 4°C, representing cold environments; 25°C, representing mesophilic environments; and 37°C, representing mammalian host body temperature.

We randomly assigned *Psychrobacter* accessions to blocks of 10 strains to be tested simultaneously (several accessions were included in multiple blocks; see Table S2). We first grew strains to saturation, washed them, diluted them to an optical density at 600 nm (OD_600_) of 0.3 in sterile phosphate-buffered saline (PBS), and inoculated them in 100 μl of medium with a final ratio of 1:1,000. We used 96-well plates, with 10 inocula in 5 replicates and 10 uninoculated medium wells per plate; all periphery wells were filled with water to reduce edge effects. Plates were incubated at each temperature to reflect all 24 growth conditions. We measured the OD_600_ (Spark plate reader; Tecan, Zürich, Switzerland) every 8 h during the first week of incubation and then every 24 h and let cultures grow until stationary phase (3 to 12 weeks).

We used the same culture dilutions from the growth assays as inocula for bile resistance assays. We spotted 10 μl of diluted culture onto both tryptic soy agar (TSA) and TSA amended with 0.5% (wt/vol) bile salt (Difco bile salts no. 3 from BD Biosciences, San Jose, CA, USA) and incubated the plates at the strains’ preferred temperatures (Table S2). We incubated the plates for 2 weeks and then scored strains for growth on bile compared to the no-bile control as either “sensitive” if no growth or “resistant” for growth similar to the control. For several strains, no growth was observed on the control plate, and these were marked as “ND” or “no data.”

*P. phenylpyruvicus* and *P. sanguinis* exhibited the inability to grow in liquid culture under the conditions tested. Hence, we first streaked 5 strains of these 2 species on Columbian blood agar which we incubated for 3 days at 37°C, and then we washed and diluted them as for the liquid cultures. We spotted dilutions onto LB or MM medium with 3% agar, supplemented with the tested salt concentrations as above, and incubated them at 4°C, 25°C, and 37°C. *P. sanguinis* strain 1501 was unable to grow on any of the base media, so we repeated the same process with medium supplemented with 0.1% Tween 80. We scored growth for all plates after 2 weeks.

For 2 accessions, we could not confirm the purity of the cultures used in the phenotypic screen and subsequently removed them from analysis. They were removed from subsequent genomic analysis as well.

### Growth probabilities.

We scored each replicate as either “growth positive,” meaning the accession grew, or else “growth negative,” if the replicate never reached a maximum OD_600_ of 0.15 over the course of the experiment. For each strain and condition (medium, salt, and temperature), we calculated a growth probability, which corresponds to the median value of growth positivity/negativity of all replicates for that condition.

For the type strains included in the phenotypic screen, we cross-checked growth probabilities at 4°C and 37°C with the published type strain descriptions where the original publications were clear about conditions tested. For the majority of strains, the type strain data and our data are in agreement; however, there are discrepancies between the type strain publications and our own data for the following strains: *P. ciconiae*, *P. fozii*, P. marincola, *P. namhaensis*, and P. submarinus. In some cases, we observed growth under temperatures where growth was previously unobserved; *P. ciconiae* and *P. marincola* have not before been reported to grow at 4°C, and *P. fozii* has not been reported to grow at 37°C. In other cases, we observed no growth in temperatures that have previously been reported to support growth; *P. submarinus* is expected to grow at 4°C and *P. namhaensis* is expected to grow at 37°C. The conditions tested in the type strain publications often differ from the conditions used in our phenotypic screen.

### Genome sequencing, assembly, and annotation.

We extracted genomic DNA from cultures grown in their preferred conditions using the Gentra Puregene tissue kit (Qiagen, Valencia, CA, USA). We initially sequenced samples using the MiSeq 2 × 250-bp and the HiSeq 2 × 150-bp paired-end read technology (Illumina, San Diego, CA, USA) as previously described (83). We constructed libraries using the Nextera DNA sample preparation kit (Illumina) with modifications: we sheared 1 ng of DNA with in-house-generated Tn*5* transposase and then amplified and barcoded it with custom primers for 7 to 14 cycles. We pooled samples and size selected using magnetic beads for MiSeq libraries or BluePippin (Sage Science, Beverly, MA, USA) for HiSeq libraries. After dilution to 4 nM for MiSeq and 2.5 nM for HiSeq, we stored libraries at −20°C until sequencing.

After sequencing and demultiplexing, we validated raw reads with fqtools (v2.0) and deduplicated them with Clumpify (v37.78, dedupe = t, dupedist = 40 for HiSeq/2500 for MiSeq, optical = t). BBDuk (v37.78) and Skewer (v0.2.2) were used to remove sequencing adapters and filter reads (minimum read length = 100 bp, minimum PHRED quality score = 25). At multiple steps throughout, we used FastQC (v0.11.7) and MultiQC (v1.7) to monitor quality. After quality control, reads were assembled *de novo*. First, we subsampled reads using seqtk (v1.3, number of subsampled reads per sample = 1,000,000), normalized by bbnorm (v37.78, target = 50, k = 31, minkmers = 15, prefilter = t), and then assembled with SPAdes (v3.12.8, cov_cutoff = off, set to “careful.” minimum scaffold length = 500 bp), followed by refinement with Pilon (v1.22, chunksize = 10,000,000). Finally, we assessed the assemblies for quality using CheckM. We assigned taxonomy using Sourmash (v2.0.0a4, scaled = 10,000, k = 31) and GTDB-Tk (v1.0.2, min_perc_aa = 10) and annotated assemblies using Prokka.

For genomes that were particularly fragmented (having greater than 250 contigs), we performed additional long-read sequencing (Table S4). We constructed Oxford Nanopore libraries using the ligation sequencing and native barcode ligation kits (Oxford Nanopore, Oxford, United Kingdom). We sequenced the libraries using the MinION system run with software MinKNOW (Oxford Nanopore). We basecalled and demultiplexed reads using ont-Guppy (v3.2.4) and used Porechop (97) (v0.2.4, adapter threshold - 90, minimum PHRED quality score = 8, minimum read length = 500 bp) to ensure that the adapters were removed, along with poor-quality reads. We generated summary statistics and summary plots and removed lambda phage reads using NanoPack (98) (nanocomp v1.11.3, nanofilt v2.7.1, nanoget v1.14.0, nanolyse v1.1.3, nanomath v0.23.3, nanoplot v1.31.0, nanostat v1.2.1). After quality control, we combined the long reads with the short (generated by the MiSeq and HiSeq libraries described above) for hybrid assembly. We assembled and analyzed the hybrid assemblies in largely the same way as the short-read assemblies described above, however, replacing SPAdes with Unicycler (99) (v0.4.8, minimum contig length = 500 bp, mode = normal).

We followed the quality control cutoffs suggested by CheckM and removed two genomes for having contamination higher than 5% and one for having completion less than 90%. We removed two additional genomes as the taxonomic classification was outside the *Psychrobacter* genus. These accessions were removed from genomic and phenotypic analysis. All further analyses use the 85 accessions which passed all quality control (QC) measures. For a full description of sequencing for each accession, see Table S4.

We annotated genomes with Prokka and eggNOG-Mapper. A phylogeny of the accessions was generated using PhyloPhlAn, with Moraxella lincolnii as an outgroup. Again, the phylogeny was visualized and annotated using iTOL. We used PanX to analyze the *Psychrobacter* pangenome and R to explore gene presence-absence data as described above with the *Moraxellaceae* family. Pseudogenes were predicted using the DFAST core workflow (100). We used treeWAS (72) to perform a pangenome-wide association study relating data collected in the phenotypic screen to genomic data.

### Microbiome diversity of polar bear feces.

We collected 86 polar bear fecal samples from several regions in Canada, including samples from 10 captive bears, fed various diets or fasted, and 76 samples from an unknown number of wild bears, feeding on unknown diets. The feces from the captive bears were forwarded from institutions within Canada and did not require permitting for their passage to Queen’s University. The captive samples comprised 5 fecal samples from a single bear sequentially fed various diets of Arctic char, harp seal, and a “zoo diet” at the Polar Bear Habitat in Cochrane during 2010; 2 samples from each of 2 bears held at the Metro Toronto Zoo, fed a consistent “zoo diet” during 2010; and a single sample from a bear held at the Churchill Polar Bear Holding Facility in Churchill during 2010, where the bears are given only water until release. We collected all wild bear feces from M’Clintock Channel and Hudson Strait in Nunavut in accordance with permits prior to shipping to Queen’s University: we collected 24 samples from M’Clintock Channel under Wildlife Research permits issued in 2007, 2008, 2009, 2010, and 2011 to Peter Van Coeverden de Groot, and 9 samples from Hudson Strait collected in 2011 under a Wildlife Research permit to Grant Gilchrest (Environment Canada). We collected 43 wild samples from the Wapusk National Park in Manitoba from 2007 to 2010 under a Canada Parks permit to Robert Rockwell (American Museum of Natural History).

We confirmed that samples collected from wild bears originated from bears by sequencing a cytochrome *b* gene fragment using metabarcoding approaches based on Ion Torrent (Ion Torrent Systems Inc., Gilford, NH, USA) and 454 pyrosequencing (454 Life Sciences, Branford, CT, USA) next-generation sequencing technologies (101). We also used this method to analyze what prey animals the polar bears had been feeding on at the time of sample deposition. We visually inspected the samples for confirmation on the dietary analysis. We extracted DNA from the fecal samples using the DNeasy blood and tissue kit (Qiagen) and characterized the gut bacterial community by amplification and sequencing of the V4 region of the 16S rRNA gene as described previously (102). We used QIIME (103) (v2, DADA2 for quality control, F read trimmed to 200 bp, R read trimmed to 110 bp, samples rarefied to 20,000 sequences) for sequence processing and Silva (138 SSURef NR99 515F/806R) to assign taxonomy.

### Isolation of *Psychrobacter* sp. from polar bear feces.

Two wild polar bear fecal samples were combined and diluted to 1 mg/ml in PBS. We plated the solution onto LB agar plus 6% NaCl and incubated it at 14°C for 10 days. A single colony grew and was identified as Psychrobacter faecalis by colony PCR and Sanger sequencing of the full-length 16S rRNA gene (described below). This isolate is designated *P. faecalis* PBFP-1 and is included in the phenotypic screen described above and the genomic analysis.

### Gnotobiotic mouse colonizations.

We selected 8 accessions of *Psychrobacter*—*P. cibarius* JG-220, *P. ciconiae*, *P. faecalis* PBFP-1, *P. immobilis* S3, *P. lutiphocae*, *P. okhotskensis* MD17, *P. namhaensis*, and *P. pacificensis*—as inocula for gnotobiotic mouse studies based on their derivation sources and phylogenetic breadth. We grew each accession under its preferred conditions (Table S3) to saturation, spun it at 10°C at 2,500 rpm for 25 min, washed it with sterile PBS, resuspended it in 15% (vol/vol) glycerol in PBS, and flash froze it in liquid N_2_. Inoculum samples were stored at −80°C until the mouse experiments were performed by animal caretakers as follows. For *P. ciconiae*, *P. faecalis*, *P. namhaensis*, and *P. pacificensis*, experiments were performed by Taconic Biosciences (Rensselaer, NY, USA) staff, while for *P. cibarius*, *P. immobilis*, and *P. okhotskensis*, experiments were performed by Max Planck Institute for Developmental Biology (MPIDB) animal caretakers. *P. lutiphocae* was included in both Taconic and MPIDB experiments.

Five- to 6-week-old germfree male C57BL/6J mice were orally inoculated with approximately 10^7^ CFU (*n* = 4 per *Psychrobacter* accession, *n* = 8 for *P. lutiphocae*). Mice were cohoused (Taconic) or single-housed (MPIDB) in sterile cages (IsoCage P; Tecniplast) and provided autoclaved water and sterile chow (NIH31M) *ad libitum*. Three weeks postcolonization, mice were sacrificed via CO_2_ asphyxiation (Taconic) or CO_2_ asphyxiation followed by cervical dislocation (MPIDB), after which cecal contents were immediately collected, flash frozen, and stored at −80°C prior to use. The Taconic experiments were performed in compliance with Taconic’s IACUC, and the MPIDB experiments were approved by and performed in accordance with the local animal welfare authority’s legal requirements.

To determine the bacterial colonization density in the mouse gastrointestinal tract, we serially diluted 2 aliquots per mouse of cecal material (50 mg each) and incubated them on plates under their preferred conditions (Table S3) for 3 to 5 days. If no colonies were observed, we spread an inoculating loop of the undiluted aliquot onto brain heart infusion agar and incubated it at 37°C for 2 weeks. For samples where we again observed no colonies, we categorized these *Psychrobacter* accessions as “nonpersistent.” For the samples that did show colony growth, we confirmed the identity of the colonies as *Psychrobacter* using Sanger sequencing of the full-length 16S rRNA gene as described below.

### 16S rRNA gene Sanger sequencing.

We prepared “colony” PCR mixtures following the protocol for Phusion high-fidelity polymerase (New England Biolabs, Ipswich, MA, USA), swirling a colony of interest in the reaction mixture as a substitute for the DNA template, and using the 27F and 1391R universal full-length 16S rRNA gene primers (104) for amplification. The reaction mixtures were incubated on the Mastercycler Pro S thermocycler (Eppendorf, Hamburg, Germany) following the Phusion protocol, using a touchdown program for the annealing temperature (dropping the annealing temperature at a rate of 1°C per cycle from 70°C to 55°C, then annealing at 55°C for 15 cycles). We cleaned the products using the DNA Clean & Concentrator-25 kit (Zymo Research, Irvine, CA, USA) and checked the concentration using the DS 11+ spectrophotometer (DeNovix, Wilmington, DE, USA). We used the cleaned DNA products as the templates for the Sanger sequencing reaction, following the ABI Prism BigDye Terminator cycle sequencing kit (ThermoFisher Scientific, Waltham, MA, USA) protocol with the 27F primer. The sequencing was performed using the 3730xl DNA analyzer (ThermoFisher). Upon sequencing completion, we performed a BLAST search (105) of the sample sequences against the NCBI nonredundant nucleotide database. We examined the top 10 hits to confirm the sample identity.

### Statistical analysis.

We performed all data processing and statistical analysis using R (106) (v3.6.2) or Python (v3.6.10). We compared means between groups using a Kruskal-Wallis test followed by a pairwise Wilcoxon rank sum when more than two groups were compared. We tested differences in frequencies between groups with more than 10 observations with a χ^2^ test, repeated 100 times with down-sampling in order to correct for sampling sizes between groups. We measured phylogenetic signal using a log-likelihood ratio test on Pagel’s λ (Pagel’s λ fitted using the phylosig function from the phytools R package [96] [v0.7-47] and the null hypothesis being λ = 0). When applicable, we tested for the confounding of phylogeny with our groups of interest using the aov.phylo function from the R package geiger (107) (v2.0.7). We adjusted all *P* values for multiple comparisons with the Benjamini-Hochberg (BH) correction method.

### Data availability.

Raw sequences for the *Psychrobacter* genome sequencing and polar bear feces 16S rRNA gene sequencing, as well as assembled *Psychrobacter* genomes, are available in the European Nucleotide Archive under the accession no. PRJEB40380. Annotated *Psychrobacter* genomes are available at ftp://ftp.tue.mpg.de/pub/ebio/dwelter. Raw data, R notebooks, and Python scripts for the analyses are available at https://github.com/dkwelter/Welter_et_al_2020.
